# Preterm Birth, Fetal Growth Restriction and Early Postnatal Body Mass Index Normalisation Predict Adult Anthropometry

**DOI:** 10.1111/apa.70368

**Published:** 2025-11-12

**Authors:** Achim Fieß, Alica Hartmann, Eva Mildenberger, Dirk Wackernagel, Julia Winter, Karl J. Lackner, Norbert Pfeiffer, Sandra Gißler, Alexander K. Schuster

**Affiliations:** ^1^ Department of Ophthalmology University Medical Center of the Johannes Gutenberg University Mainz Mainz Germany; ^2^ Division of Neonatology, Department of Pediatrics University Medical Center of the Johannes Gutenberg‐University Mainz Mainz Germany; ^3^ Institute of Clinical Chemistry and Laboratory Medicine University Medical Center of the Johannes Gutenberg University Mainz Mainz Germany

**Keywords:** adult anthropometry, fetal growth restriction, metabolic outcomes, postnatal body mass index, prematurity

## Abstract

**Aim:**

This study assessed the long‐term impact of prematurity and fetal growth restriction on adult anthropometric and metabolic outcomes.

**Methods:**

In this retrospective cohort study at the University Medical Center Mainz, Germany, adults aged 18–52 years born between 1969 and 2002 were examined between 2019 and 2021. Participants were classified by gestational age and birth weight percentiles as small (SGA), appropriate (AGA), or large for gestational age (LGA). Adult anthropometric and metabolic parameters were analysed using multivariable regression models in relation to gestational age, fetal growth, and early postnatal BMI normalisation.

**Results:**

Adults born preterm were shorter, lighter and had smaller head circumferences in adulthood than those born at term. Moderately to severely SGA individuals were shorter and lighter and had smaller head circumferences and lower BMI. Participants born LGA were taller and heavier than individuals born AGA. Early postnatal BMI normalisation was associated with improved adult anthropometry in preterm SGA participants only. No significant associations were observed between gestational age or birth weight groups and metabolic outcomes, including metabolic syndrome, hypertension, cholesterol, glucose and triglycerides.

**Conclusion:**

Prematurity and fetal growth restriction were linked to differences in adult anthropometry, whereas early postnatal BMI normalisation appeared beneficial in preterm SGA individuals.

**Abbreviations**
AGA: appropriate for gestational age
BMI: body mass index
LGA: large for gestational age
SGA: small for gestational age

**Summary**

- Evidence on how prematurity and restricted fetal growth affect adult anthropometry and metabolic health has remained limited, prompting this study.
- This study of 610 adults showed that both prematurity and being born small for gestational age were associated with smaller adult body anthropometry, while no links to adverse metabolic outcomes were detected.
- Early postnatal normalisation of body mass index improved adult anthropometry in participants born preterm and small for gestational age.

## 1 Introduction

Adverse birth timing and suboptimal fetal growth have lifelong effects on adult health. Early identification of at‐risk groups is important for clinical care and public health. Both prematurity and fetal growth restriction have significant implications for anthropometric outcomes [1] and critically influence metabolic health [2, 3, 4]. Moreover, individuals born preterm are predisposed to a range of metabolic irregularities later in life [5].

Previous studies reported that individuals born preterm or small for gestational age (SGA) were shorter in adulthood and had smaller head circumferences. Body mass index (BMI) in these individuals tended to align with that of term‐born peers during adolescence [1, 6, 7, 8, 9, 10, 11]. Evidence from earlier studies indicated that postnatal catch‐up growth may mitigate the potential consequences of prematurity or being born SGA [10, 11]. However, much of the literature used low birth weight as a proxy for fetal growth restriction and therefore failed to distinguish gestational age from fetal growth. This study addressed that methodological gap by separately analysing gestational age and fetal growth categories across the full spectrum, including individuals born large for gestational age (LGA). It also examined whether the presence and timing of postnatal BMI normalisation influenced adult phenotypes. We also performed sensitivity analyses adjusting for maternal anthropometry to help separate familial and constitutional influences from intrauterine factors.

Grounded in the Developmental Origins of Health and Disease framework [12], we hypothesised several directional associations. It was expected that lower gestational age and lower birth weight percentiles, reflecting moderate to severe SGA, would predict poorer adult anthropometry. This included shorter height, lower body weights and smaller head circumferences. In contrast, being LGA was expected to predict the opposite pattern. It was further hypothesised that gestational age and fetal growth categories would show minimal or reduced associations with adult BMI, reflecting age‐related convergence. Among participants born SGA, postnatal BMI normalisation within the first 2 years of life was expected to be associated with more favourable adult anthropometry. This included taller height, higher body weights within normative ranges and larger head circumferences compared with absent or later normalisation. Lower gestational age and being born SGA were also hypothesised to be associated with less favourable adult metabolic profiles. These were expected to include higher risks of metabolic syndrome, hypertension and elevated fasting lipids or glucose, partly independent of age, sex and maternal anthropometry.

Based on these hypotheses, the study aimed to quantify associations of gestational age with adult anthropometric and metabolic outcomes. Additional aims were to assess the effects of fetal growth categories on adult anthropometric and metabolic outcomes. The study also examined whether the presence and timing of postnatal BMI normalisation influenced these outcomes.

## 2 Materials and Methods

**FIGURE 1:**
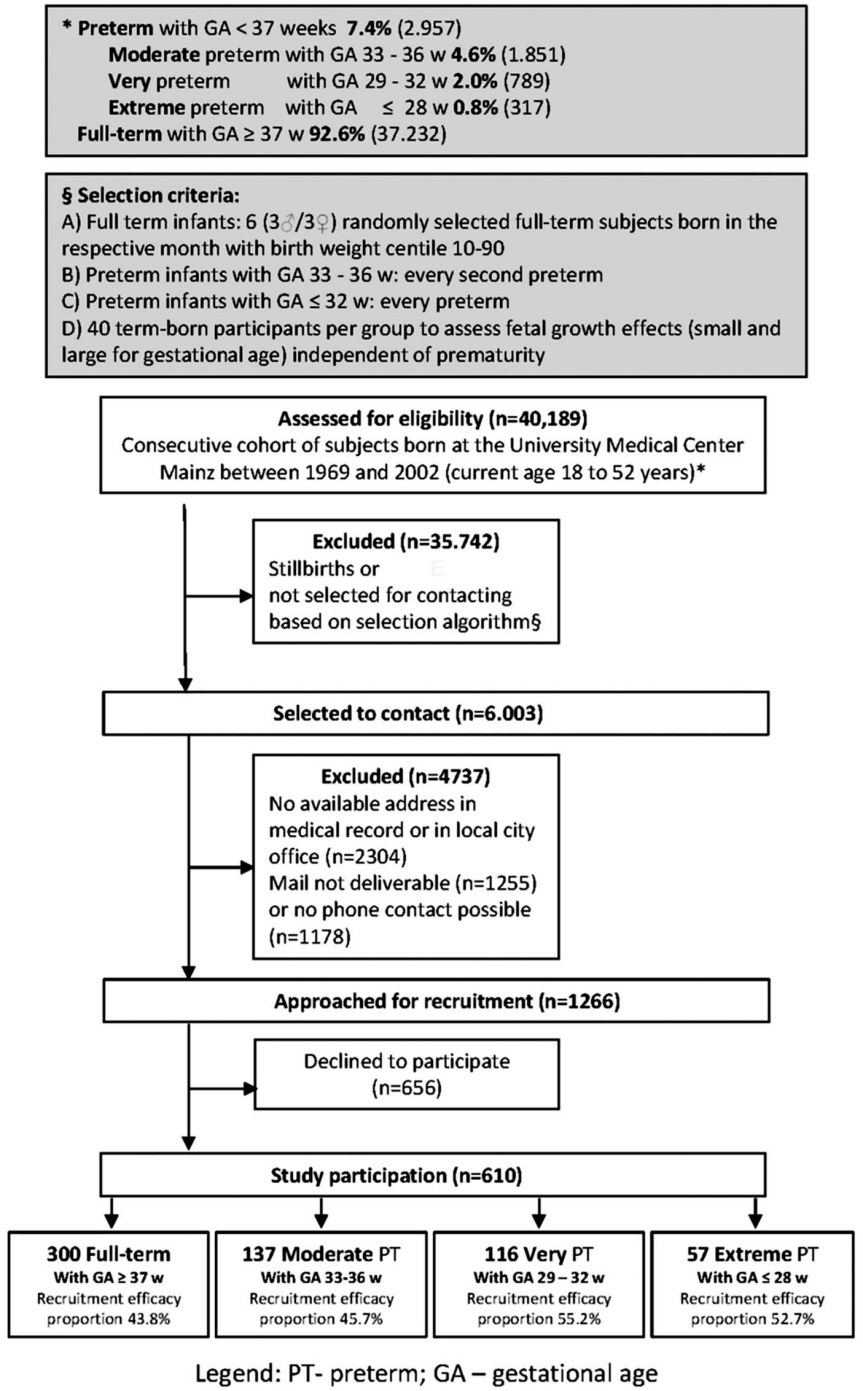
Flowchart of study inclusion and exclusion criteria.

This was a retrospective cohort study conducted at the University Medical Center Mainz, Germany. Participants were born either preterm or at term between 1969 and 2002 and were aged 18–52 years at enrolment. The design combined retrospective cohort elements with the prospective acquisition of follow‐up data. Every second randomly selected preterm infant with a gestational age of 33–36 weeks was invited to participate. In addition, all preterm newborns with a gestational age of 32 weeks or less were included. For the term‐born comparison group (*n* = 300), a month‐year based sampling algorithm was applied. For each month between January 1969 and December 2002, six eligible candidates were drawn. Three females and three males with birth weights between the 10th and 90th percentiles were invited sequentially. In addition, four term‐born birth weight strata were recruited to examine fetal growth independent of prematurity. Each group included 40 participants classified as severely SGA, moderately SGA, moderately LGA or severely LGA, resulting in a total of 300 term‐born participants. Recruitment outcomes, including participants who were approached, unreachable or declined to take part, are shown in Figure 1.

Participants were categorised by gestational age into four groups. The term group included 300 participants born at 37 + 0 weeks or later. The moderate preterm group included 137 participants born between 33 + 0 and 36 + 6 weeks. The very preterm group included 116 participants born between 29 + 0 and 32 + 6 weeks and the extremely preterm group included 57 participants born before 29 + 0 weeks. Comprehensive medical exams and personal interviews were conducted between 2019 and 2021.

### 2.1 Assessment of Prenatal and Postnatal History

The participants' prenatal and postnatal histories were obtained from medical records. Extracted data included gestational age in weeks, birth weight in kilograms, placental insufficiency, maternal smoking, preeclampsia, breastfeeding and perinatal adverse events. Their birth‐weight percentiles were calculated according to Voigt et al. [13].

### 2.2 Anthropometric and Metabolic Parameters in Adulthood

Characteristics recorded for the study population included age, sex, body height, body weight, BMI and head circumference. Arterial hypertension was defined as the use of antihypertensive medication, systolic blood pressure above 140 mmHg, diastolic blood pressure above 90 mmHg or a documented diagnosis. Total cholesterol, high‐density lipoprotein, low‐density lipoprotein, triglycerides and fasting glucose levels were measured using standard clinical chemistry methods on the Abbott Alinity c system manufactured by Abbott Diagnostics in Abbott Park, Illinois, USA. Metabolic syndrome was defined by the National Cholesterol Education Program Adult Treatment Panel III criteria [14]. Abdominal obesity was defined as a waist circumference greater than 102 cm in men and greater than 88 cm in women. Additional components included triglyceride levels of at least 150 mg/dL and high‐density lipoprotein cholesterol below 40 mg/dL in men or below 50 mg/dL in women. Blood pressure of 135/85 mmHg or higher and fasting glucose above 110 mg/dL or the presence of type 2 diabetes were also part of the definition.

### 2.3 Postnatal BMI Normalisation

Anthropometric parameters were obtained from routine paediatric screening examinations conducted in Germany as part of the national child health programme. This programme is regulated by the Federal Joint Committee [15]. Percentiles for each infant's measurements were calculated with German reference data. For birth, the birth weight percentile by gestational age was calculated using Voigt data [13]. For subsequent time points, BMI percentiles were calculated using reference data from the German Health Interview and Examination Survey for Children and Adolescents [16]. These percentiles were calculated using the *childsds* package in R, based on data from Kromeyer‐Hauschild et al. [17]. In the present study, we used BMI percentiles as a proxy for postnatal growth trajectories. Specifically, we examined postnatal BMI normalisation, defined as an increase of BMI above the 10th percentile for infants born SGA, or a decline into the 10th–90th percentile range for infants born LGA. We evaluated BMI normalisation within the first 2 years of life, based on prior evidence that most infants with growth restriction normalise during this period [18].

### 2.4 Covariates

The following covariates were considered as potential factors influencing adult outcomes. Gestational age deficit was defined as the number of weeks shorter than the standard 40‐week term and abnormal fetal growth referred to being born SGA or LGA.

### 2.5 Statistical Analysis

For descriptive purposes, we calculated absolute and relative frequencies for dichotomous variables and means and standard deviations were calculated for normally distributed variables. Analysis of Variance was used to compare lab parameters across gestational age and birth weight percentile groups, calculating global *p*‐values to identify any significant mean differences between them. Multivariable linear regression was performed to analyse the associations of gestational age and abnormal fetal growth with adult body height and head circumference, adjusting for age and sex. Quantile regression at the median, where tau equals 0.5, was used to assess associations with adult weight and BMI. This approach was chosen because residual diagnostics showed skewed distributions that violated the assumptions of linear regression. Associations between metabolic parameters and gestational age or abnormal fetal growth were analysed using linear regression. Logistic regression was applied for binary metabolic outcomes such as hypertension and metabolic syndrome.

We also adjusted for maternal anthropometry in the sensitivity analysis and investigated interactions between birth weight percentile groups and gestational age deficit. The association between postnatal BMI normalisation after 2 years and adult anthropometric outcomes was analysed using multivariable linear and quantile regression. Participants were compared according to whether or not BMI normalisation had occurred. The association between the timing of BMI normalisation and adult anthropometric parameters was analysed using a separate linear model. Early normalisation was defined as within the first 6 months of life and later normalisation as between six and 48 months. This was an explorative study with no adjustment for multiple testing. All analyses were performed in R version 4.3.2 provided by the R Foundation for Statistical Computing in Vienna, Austria [19].

### 2.6 Ethics

All participants provided written informed consent in accordance with the International Council for Harmonisation Guideline for Good Clinical Practice and the German Society for Epidemiology recommendations for Good Epidemiological Practice, as well as the Declaration of Helsinki. The study protocol was approved by the ethics committee of the Medical Chamber of Rhineland‐Palatinate under reference number 2019–14161. The original approval was granted on 29 May 2019 and last updated on 2 April 2020.

## 3 Results

### 3.1 Participant Characteristics

This study involved 610 (53.9% women) participants born preterm and term at an average age of 29.01 ± 8.93 years. A total of 1266 eligible individuals were invited, of whom 656 declined to participate, resulting in a participation rate of approximately 50%. It should be noted that there was only a small number of LGA births in the preterm groups. Table 1 highlights notable differences in birth weight, perinatal complications and neonatal outcomes across gestational age groups. As expected, both birth weight and birth weight percentiles decreased with lower gestational age, with the most extreme preterm group at 28 weeks or less showing the lowest values. The prevalence of SGA infants was higher in the preterm groups, while LGA cases were rare in the lowest gestational age categories. Maternal risk factors such as preeclampsia, placental insufficiency and smoking were more frequent in preterm births. Breastfeeding rates declined with decreasing gestational age. Significant anthropometric differences across gestational age groups are presented in Table 2. Table 3 details the anthropometric and metabolic characteristics by birth weight percentiles. No significant differences in metabolic parameters were found between gestational age and metabolic syndrome, hypertension, high‐density lipoprotein cholesterol, fasting glucose, triglycerides or creatinine. Total cholesterol levels were slightly higher in the moderately preterm group compared with the term group. In the other preterm groups cholesterol levels were lower. No associations were observed between metabolic parameters and birth weight percentile groups.

**TABLE 1:** Characteristics of the study sample (*n* = 610) of the Gutenberg Prematurity Study.

	Group 1	Group 2	Group 3	Group 4
	GA ≥ 37 weeks	GA 33–36 weeks	GA 29–32 weeks	GA ≤ 28 weeks
Number of participants	300	137	116	57
Female participants	159 (53.0%)	82 (59.9%)	60 (51.7%)	28 (49.1%)
Age (years)	30.10 ± 9.42	29.47 ± 9.2	27.88 ± 8.0	24.51 ± 5.5
Birth weight (g)	3436 ± 901	2068 ± 464	1500 ± 364	855 ± 215
Birth weight percentile	49.51 ± 36.96	25.25 ± 24.15	43.09 ± 26.42	37.60 ± 24.77
Severe SGA (BW percentile < 3)	40 (13.3%)	20 (14.6%)	4 (3.4%)	1 (1.8%)
Moderately SGA (BW percentile 3 to < 10)	40 (13.3%)	26 (19.0%)	6 (5.2%)	9 (15.8%)
AGA (BW percentile 10–90)	140 (46.7%)	88 (64.2%)	104 (89.7%)	47 (82.5%)
Moderately LGA (BW percentile > 90 to 97)	40 (13.3%)	1 (0.7%)	2 (1.7%)	0 (0.0%)
Severe LGA (BW percentile > 97)	40 (13.3%)	2 (1.5%)	0 (0.0%)	0 (0.0%)
Gestational age (weeks)	39.23 (1.50)	34.31 ± 0.95	30.47 ± 1.16	26.32 ± 1.38
(min‐max)	(37–43)	(33–36)	(29–32)	(23–28)
Preeclampsia (yes)	32 (10.7%)	24 (17.5%)	16 (13.8%)	11 (19.3%)
Placental insufficiency (yes)	13 (4.3%)	16 (11.7%)	3 (2.6%)	3 (5.3%)
Maternal smoking (yes)	13 (4.3%)	8 (5.8%)	9 (7.8%)	8 (14.0%)
HELLP syndrome (yes)	1 (0.3%)	6 (4.4%)	2 (1.7%)	3 (5.3%)
Gestational diabetes (yes)	5 (1.7%)	7 (5.1%)	2 (1.7%)	1 (1.8%)
Breastfeeding (yes)	172 (57.3%)	75 (54.7%)	59 (50.9%)	25 (43.9%)
**Maternal characteristics**				
Number of mothers	141	63	47	27
Age (years)	61.00 ± 7.79	57.89 ± 6.72	58.36 ± 6.28	57.63 ± 5.38
Body height, mean (SD)	165.20 ± 15.25	161.87 ± 21.49	168.78 ± 7.17	159.39 ± 31.99
Body weight, mean (SD)	75.62 ± 15.96	77.01 ± 20.77	74.54 ± 13.78	73.17 ± 15.35
Body‐mass‐index (BMI), mean (SD)	27.14 ± 5.33	28.03 ± 6.48	26.28 ± 6.03	26.30 ± 5.14

Abbreviations: AGA, appropriate for gestational age; BW, birth weight; LGA, large for gestational age; SGA, small for gestational age.

**TABLE 2:** Anthropometric and biochemical data in adulthood stratified by gestational age.

	Group 1	Group 2	Group 3	Group 4	*p* *
	GA ≥ 37 weeks	GA 33–36 weeks	GA 29–32 weeks	GA ≤ 28 weeks	
Number of Participants	300	137	116	57	
Body height, mean (SD)	174.49 (10.67)	171.18 (9.42)	171.91 (9.46)	168.30 (8.83)	< 0.001
Body weight, mean (SD)	77.35 (20.71)	73.76 (17.43)	72.56 (19.58)	66.62 (18.35)	0.001
Body‐mass‐index (BMI), mean (SD)	25.23 (5.56)	25.17 (5.12)	24.45 (5.82)	23.18 (4.78)	0.08
Head circumference, mean (SD)	56.27 (3.91)	55.73 (2.17)	55.46 (2.19)	54.92 (4.57)	0.02
Metabolic syndrome (yes), defined by NCEP‐ATP III criteria	35 (11.7%)	18 (13.1%)	12 (10.3%)	3 (5.3%)	0.44
Hypertension (yes)	83 (27.7%)	40 (29.2%)	34 (29.3%)	15 (26.3%)	0.94
LDL‐cholesterol, mean (SD) (mg/dL)	105.89 (30.96)	110.73 (31.59)	104.41 (29.63)	95.65 (22.65)	0.04
HDL‐cholesterol, mean (SD) (mg/dL)	57.19 (13.17)	58.66 (13.74)	56.18 (11.77)	56.40 (13.37)	0.41
Cholesterol, mean (SD) (mg/dL)	185.11 (35.66)	192.36 (37.81)	182.14 (35.66)	176.02 (34.88)	0.04
Fasting glucose, mean (SD) (mg/dL)	95.34 (30.89)	95.17 (31.33)	94.62 (13.76)	90.34 (16.87)	0.75
Triglycerides, mean (SD) (mg/dL)	110.44 (58.89)	114.89 (59.84)	108.03 (50.81)	111.73 (70.77)	0.92
Creatinine, mean (SD) (mg/dL)	0.77 (0.16)	0.80 (0.21)	0.76 (0.14)	0.75 (0.14)	0.16

Abbreviation: GA, gestational age (weeks).

* ANOVA was performed to compare differences between the gestational age groups, and global *p*‐values were computed to assess whether there is at least one significant difference in means across any of the groups.

**TABLE 3:** Anthropometric and biochemical data in adulthood stratified by birth weight percentiles.

	Severely SGA	Moderately SGA	AGA	Moderately LGA	Severely LGA	*p* *
	< 3 percentile	3 to < 10 percentile	10–90 percentile	> 90 to 97 percentile	> 97 percentile	
Number of Participants	65	81	379	43	42	
Body height, mean (SD)	168.25 (9.34)	171.28 (9.69)	172.33 (9.72)	178.95 (9.57)	179.36 (11.68)	< 0.001
Body weight, mean (SD)	69.04 (16.33)	72.41 (18.20)	73.37 (18.54)	85.57 (23.81)	88.51 (24.75)	< 0.001
Body‐mass‐index (BMI), mean (SD)	24.62 (4.96)	24.58 (5.45)	24.56 (5.21)	26.57 (6.76)	27.03 (6.47)	0.01
Head circumference, mean (SD)	54.94 (2.15)	55.80 (2.21)	55.67 (3.84)	57.50 (2.15)	57.65 (2.33)	< 0.001
Metabolic syndrome (yes), defined by NCEP‐ATP III criteria	6 (9.2%)	5 (6.2%)	45 (11.9%)	6 (14.0%)	6 (14.3%)	0.52
Hypertension (yes)	18 (27.7%)	19 (23.5)	108 (28.5%)	13 (30.2)	12 (28.6%)	0.91
LDL‐ cholesterol, mean (SD)	106.39 (34.64)	99.77 (31.99)	106.45 (29.93)	109.19 (31.25)	107.15 (21.93)	0.55
HDL‐ cholesterol, mean (SD)	57.45 (12.67)	57.28 (14.66)	57.63 (12.93)	54.09 (12.74)	56.11 (11.39)	0.72
Cholesterol, mean (SD)	187.18 (39.36)	181.37 (35.83)	186.01 (36.86)	184.72 (32.53)	184.37 (30.50)	0.92
Fasting glucose, mean (SD)	93.26 (15.29)	98.35 (42.53)	94.62 (26.86)	93.55 (9.19)	90.75 (13.76)	0.76
Triglycerides, mean (SD)	116.93 (70.66)	122.53 (63.89)	108.75 (56.44)	107.47 (53.14)	105.81 (56.19)	0.4
Creatinine, mean (SD)	0.79 (0.16)	0.80 (0.14)	0.76 (0.18)	0.84 (0.14)	0.70 (0.14)	0.01

Abbreviations: AGA, appropriate for gestational age; LGA, large for gestational age; SGA, small for gestational age.

* ANOVA was performed to compare differences between the birth weight percentile groups and global *p*‐values were computed to assess whether there is at least one significant difference in means across any of the groups.

### 3.2 Univariable and Multivariable Analyses

Associations between gestational age and adult anthropometric outcomes were explored. Participants born at lower gestational ages showed reduced adult height, lower body weights and smaller head circumferences (Table 4). There was no association between BMI and gestational age. The association between gestational age deficit and adult height was modified by the severity of being born SGA, with a stronger effect observed among those with lower birth weight percentiles (*B* = −2.140, 95% CI −1.280 to −0.055, *p* = 0.03) (Table S1).

**TABLE 4:** Association analyses of the anthropometric parameters for adults born preterm and term (*n* = 610).

	All models adjusted for age, sex	
Body height (cm)		
Linear regression	Estimate (95% CI)	*p*
Gestational age deficit (weeks)*	−0.429 (−0.561, −0.296)	< 0.001
Severely SGA (< 3)	−5.516 (−7.347, −3.684)	< 0.001
Moderately SGA (3 to < 10)	−3.492 (−5.162, −1.822)	< 0.001
Moderately LGA (> 90 to 97)	1.809 (−0.491, 4.108)	0.12
Severely LGA (> 97)	3.192 (0.853, 5.529)	0.01

Body weight (kg)		
Quantile regression	β_(_τ_50)_ (95% CI)	*p*
Gestational age deficit (weeks)	−0.383 (−0.835, −0.162)	0.02
Severely SGA (< 3)	−4.796 (−8.600, −2.969)	0.002
Moderately SGA (3 to < 10)	−4.816 (−9.107, −1.106)	0.01
Moderately LGA (> 90 to 97)	4.871 (−1.177, 8.562)	0.08
Severely LGA (> 97)	8.164 (1.747, 11.272)	0.002

Body‐mass‐index (BMI)		
Quantile regression	β_(_τ_50)_ (95% CI)	*p*
Gestational age deficit (weeks)	−0.039 (−0.090, 0.046)	0.34
Severely SGA (< 3)	−0.243 (−1.273, 0.593)	0.67
Moderately SGA (3 to < 10)	−0.555 (−1.039, 0.393)	0.27
Moderately LGA (> 90 to 97)	0.163 (−0.091, 1.331)	0.67
Severely LGA (> 97)	0.844 (−0.046, 2.346)	0.25

Head circumference		
Linear regression	Estimate (95% CI)	*p*
Gestational age deficit (weeks)	−0.076 (−0.137, −0.016)	0.01
Severely SGA (< 3)	−0.965 (−1.805, −0.125)	0.02
Moderately SGA (3 to < 10)	−0.292 (−1.053, 0.469)	0.45
Moderately LGA (> 90 to 97)	0.972 (−0.076, 2.021)	0.07
Severely LGA (> 97)	1.217 (0.140, 2.293)	0.03

Abbreviations: AGA, appropriate for gestational age; LGA, large for gestational age; SGA, small for gestational age.

* Gestational age deficit represents the number of weeks by which the gestation is shorter than the standard term pregnancy of 40 weeks.

Severe growth restriction and moderate growth restriction were associated with a shorter body height, while those born severely LGA demonstrated greater body height in adulthood (Table 4). Participants born severely or moderately SGA had lower adult body weights, while those from the severe LGA group showed higher adult body weights. There were no associations between BMI and birth weight percentile groups. Participants born severely SGA had smaller head circumferences, while those classified as severely LGA had larger head circumferences in adulthood.

A fundamental increase in anthropometric parameters was observed with higher gestational age and higher birth weight percentile (Figure S1). Detailed data are provided in Figure S2. To improve statistical power, we calculated additional models using birth weight percentile as a continuous variable. These sensitivity analyses showed results comparable to the categorical approach (Table S2).

No significant associations were found between gestational age, birth weight percentile groups and metabolic outcomes. These included metabolic syndrome, hypertension, high‐density lipoprotein cholesterol, fasting glucose, triglycerides and total cholesterol. Participants born severely LGA had lower creatinine levels than those in the AGA reference group (Tables S3 and S4).

In addition, the multivariable regression model was adjusted for maternal height, body weight and BMI. Results shown in Table S5 revealed that participants' anthropometric measures were associated with maternal anthropometry. Associations were found between gestational age, moderate to severe growth restriction and adult height. In the case of adult body weight, there was an association with being born SGA. The previously described association with gestational age was not evident. Furthermore, a new association was observed between being moderately to severely SGA and having a lower BMI in adulthood. It should be noted that maternal anthropometric data were only available for approximately half of the participants.

**FIGURE 2:**
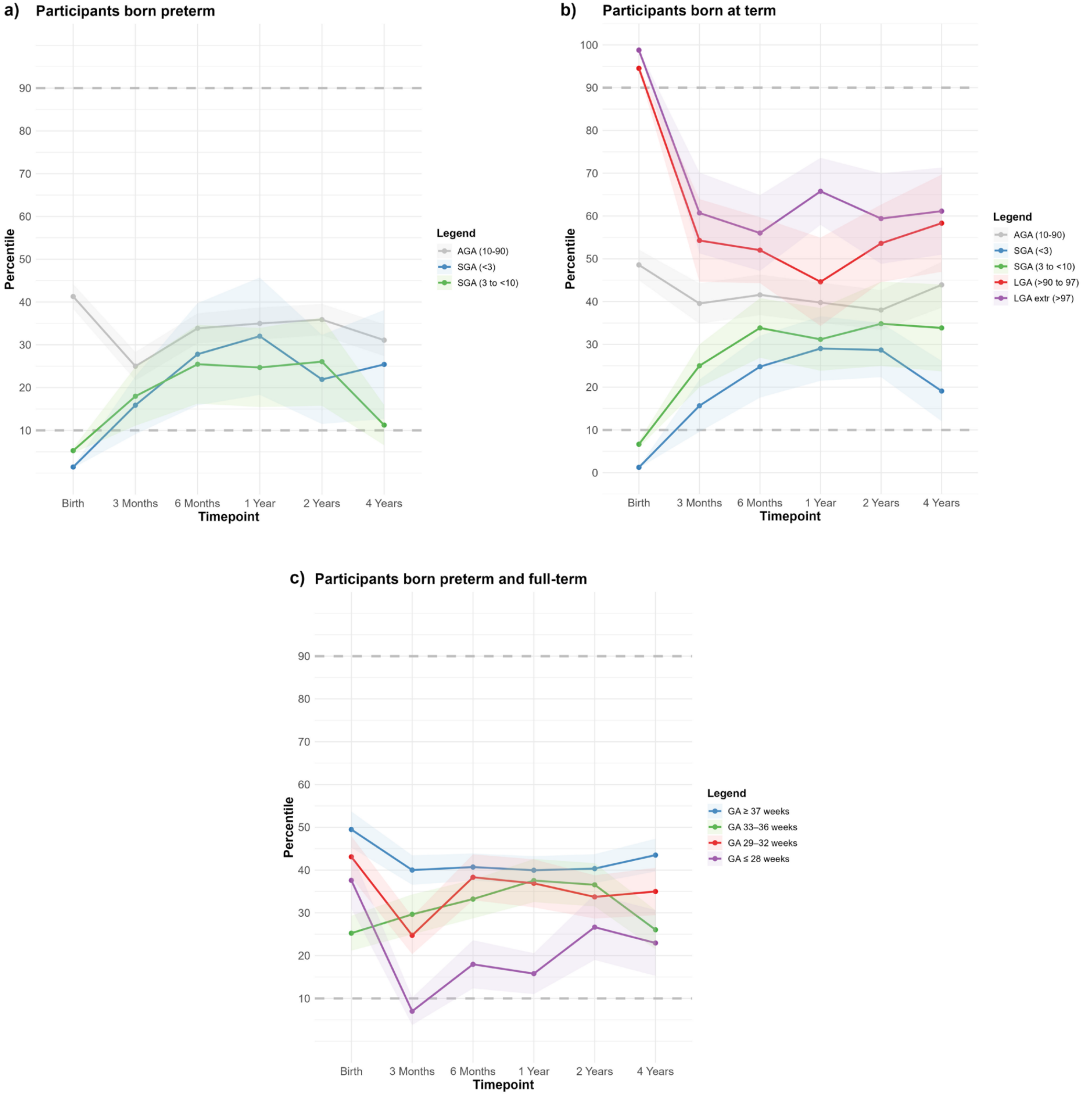
BMI percentiles over 4 years in (a) small for gestational age (SGA) and appropriate for gestational age (AGA) participants, born preterm; (b) SGA, AGA and large for gestational age (LGA) participants, born term and (c) gestational age groups, preterm and term. The transparent shaded areas represent 95% confidence intervals.

Figure 2 provides an overview of BMI percentile trajectories during the first 4 years of life, stratified by gestational age and SGA or LGA categories. Preterm infants born severely or moderately SGA showed rising BMI percentiles during the first year, while preterm AGA infants remained stable. Among term‐born infants, those born SGA showed gradual increases in BMI percentiles, whereas LGA infants demonstrated clear declines toward the AGA range. Across gestational age categories, higher gestational age was associated with more stable trajectories, while the extremely preterm group showed the greatest variability.

Preterm and SGA participants who achieved BMI normalisation within the first 2 years were taller and heavier in adulthood. They also had larger head circumferences compared with those without postnatal BMI normalisation (Figure S3a and Table 5). No such association was observed in term‐born SGA participants (Figure S3b and Table 5). Early BMI normalisation during the first 6 months of life in preterm SGA participants was associated with larger adult head circumferences compared with later BMI normalisation (Table S6). There were no differences in other anthropometric parameters (Tables S6 and S7).

**TABLE 5:** Association analyses of postnatal body mass index normalisation for adults born SGA and preterm or term (*n* = 144).

All multivariable models adjusted for age, sex				
Individuals born preterm and SGA (*n* = 66)			Individuals born at term and SGA (*n* = 80)	
Body height (cm)				
Linear regression	Estimate (95% CI)	*p*	Estimate (95% CI)	*p*
Postnatal BMI normalisation (yes)	7.439 (2.923, 11.956)	0.002	1.333 (−2.573, 5.241)	0.50
Gestational age deficit (weeks)	−0.258 (−1.018, 0.502)	0.49	−0.136 (−1.515, 1.244)	0.84
Severely SGA (< 3)	−4.373 (−8.423, −0.323)	0.04	0.767 (−2.954, 4.488)	0.68

Body weight (kg)				
Quantile regression	β_(_τ_50)_ (95% CI)	*p*	β_(_τ_50)_ (95% CI)	*p*
Postnatal BMI normalisation (yes)	18.750 (3.787, 26.975)	0.004	1.167 (−3.060, 6.624)	0.54
Gestational age deficit (weeks)	0.063 (−1.124, 2.943)	0.96	−0.778 (−2.240, 2.096)	0.35
Severely SGA (< 3)	−4.625 (−11.609, 20.379)	0.50	−0.500 (−7.314, 5.678)	0.84

Body‐mass‐index (BMI)				
Quantile regression	β_(_τ_50)_ (95% CI)	*p*	β_(_τ_50)_ (95% CI)	*p*
Postnatal BMI normalisation (yes)	1.937 (1.617, 4.136)	0.23	1.328 (−0.514, 2.318)	0.21
Gestational age deficit (weeks)	−0.158 (−0.179, 0.994)	0.63	−0.362 (−1.104, 0.263)	0.37
Severely SGA (< 3)	0.353 (−2.140, 4.393)	0.88	−0.031 (−3.079, 2.432)	0.98

Head circumference				
Linear regression	Estimate (95% CI)	*p*	Estimate (95% CI)	*p*
Postnatal BMI normalisation (yes)	2.220 (0.758, 3.682)	0.004	0.493 (−0.576, 1.561)	0.36
Gestational age deficit (weeks)	−0.168 (−0.414, 0.078)	0.17	0.061 (−0.319, 0.440)	0.75
Severely SGA (< 3)	−1.203 (−2.513, 0.108)	0.07	−0.790 (−1.831, 0.250)	0.13

Abbreviations: BMI, body mass index; CI, confidence interval; SGA, small for gestational age.

## 4 Discussion

There were three key findings in this cohort of 610 adults born preterm or at term. First, gestational age and birth weight percentiles were associated with adult anthropometric measures, confirming that prematurity and growth restriction are linked to smaller adult anthropometry. Second, postnatal BMI normalisation was beneficial for adult anthropometry in preterm SGA participants, but not in term SGA individuals. Third and most importantly, we found no significant associations of gestational age or fetal growth categories with metabolic outcomes in early adulthood.

Contrary to expectations based on the Developmental Origins of Health and Disease hypothesis [12, 20], no associations of prematurity or fetal growth restriction with metabolic syndrome, hypertension, or serum lipids and glucose were detected. This contrasts with studies reporting elevated blood pressure and impaired glucose regulation in SGA individuals during childhood or adolescence [3, 21], but is consistent with other cohorts of young adults where metabolic risks were not yet evident [22]. The relatively young mean age of participants, about 29 years, may have partly explained this discrepancy. Metabolic alterations often appear later in life. These findings suggest that fetal growth restriction and prematurity do not necessarily translate into early adult metabolic disease. Continued follow‐up is essential to determine whether risks develop with advancing age.

In line with previous registry and cohort studies [6, 23, 24, 25], lower gestational age was associated with reduced adult height. In a Swedish birth register study of more than 200 000 women, very preterm birth defined as less than 32 weeks, was linked to a reduction in adult stature of 1–2 cm compared to moderately preterm and term controls [23]. Similar findings were reported from Finnish and Norwegian cohorts restricted to very low birth weight infants < 1500 g, where adult participants at 36 years remained shorter and lighter than controls [6]. Studies in children and adolescents also consistently observed reduced stature among those born preterm [24, 25]. In addition, individuals born moderately or severely SGA were shorter and lighter in adulthood. This finding was consistent with long‐term cohort data demonstrating that reduced birth weight is associated with shorter adult height [26, 27, 28, 29], whereas high birth weight confers greater stature and higher adult body weight [30, 31]. The present study adds to this literature by simultaneously considering gestational age and fetal growth categories. Both prematurity and being born SGA independently predicted adult anthropometry. The finding of the association with gestational age was most pronounced in SGA participants. This suggests that prematurity exacerbates the long‐term impact of restricted fetal growth. Head circumference in adulthood also reflected early growth, with smaller values in preterm and SGA participants and larger values in those born LGA. This supports reports that restricted intrauterine growth limits head growth trajectories, which are known to predict later brain volume and neurodevelopment [7, 32]. The current findings extend this evidence by demonstrating that differences persist into adulthood, highlighting the potential for lasting neurodevelopmental implications of fetal growth extremes.

Jussinniemi et al. studied [6] 137 preterm participants with very low birth weights and 158 term‐born controls from Finnish and Norwegian cohorts. They observed that adults born preterm weighing under 1500 g were shorter on average at 36 years of age. Also, children and adolescents born preterm tended to be shorter in height than participants born at term.

In addition, our findings revealed that participants born severely or moderately SGA were shorter in adulthood. This was consistent with other studies showing that lower birth weight was associated with shorter adult height, whereas higher birth weight was linked to greater stature [26, 27, 28, 29]. These studies considered birth weight, not percentiles. Our regression analysis showed that both low gestational age and being born SGA were significantly associated with adult height. The association of gestational age with height was most pronounced in those born severely SGA, indicating a significant effect modification.

Preterm SGA participants who experienced BMI normalisation within the first 2 years of life were taller, heavier and had larger head circumferences in adulthood compared with those without BMI normalisation. No such associations were observed among term‐born SGA participants. Comparable findings have been reported for extremely preterm children reaching adult height similar to term‐born controls if postnatal catch‐up growth occurred [10, 25]. The absence of associations in term SGA individuals suggests that physiological responses to early growth restriction may differ between preterm and term births. Early BMI normalisation within the first 6 months of life in preterm SGA participants was associated with larger adult head circumference. This finding underscored the potential importance of early growth velocity for neurodevelopment.

Adjustment for maternal anthropometry confirmed that both gestational age and SGA remained associated with adult height, whereas lower adult weight and BMI were more strongly related to being born SGA than to prematurity. These findings align with previous observations that parental size influences offspring height more than weight [33], while SGA birth exerts long‐lasting effects on body composition across the lifespan [34, 35, 36].

Taken together, these results indicate that while the anthropometric consequences of prematurity and fetal growth restriction are consistent and robust, adverse metabolic outcomes were not apparent in early adulthood. This has clinical relevance, as it suggests that young adults born preterm or SGA may not yet manifest the metabolic risks predicted by the Developmental Origins of Health and Disease hypothesis. Nevertheless, long‐term monitoring remains warranted. Preventive strategies should continue to support healthy growth trajectories, particularly in preterm SGA individuals who appear to benefit most from early BMI normalisation.

### 4.1 Strengths and Limitations

This single‐center, hospital‐based cohort study had limitations including potential selection bias and a predominantly white participant pool limiting generalisability. Approximately 50% of invited individuals declined to participate, which may have introduced additional selection bias. It was exploratory with no corrections for multiple testing and incomplete early anthropometric data for some participants. A further limitation was that postnatal development was assessed using BMI percentiles rather than direct measures of somatic growth, meaning that our approach reflected normalisation of BMI as a proxy for early growth patterns. Despite these constraints, it was one of the most comprehensive examinations of adults born preterm across different gestational age categories and growth patterns. The study thoroughly reviewed perinatal records, followed standardised measurement procedures and maintained investigator blinding to participants' birth characteristics.

## 5 Conclusion

This study showed associations between prematurity, fetal growth characteristics and adult anthropometry. Participants born at lower gestational age and severely SGA were at high risk for a significant reduction in adult height. Furthermore, the findings highlighted the crucial role of postnatal BMI normalisation within the first 2 years of life for preterm SGA infants. This early normalisation was associated with greater adult body height, body weight and head circumference, which was not observed in term SGA participants. This suggests a nuanced interplay between gestational age, birth weight classification and postnatal growth patterns. By examining these complex interactions, our research highlights their lasting associations with adult physical development.

## Author Contributions

Prof. Achim Fieß and Prof. Alexander K. Schuster conceptualised and designed the study and drafted the initial manuscript. Alica Hartmann carried out the initial analyses and drafted the initial manuscript. Dr. Sandra Gißler also assisted in drafting the initial manuscript. All authors analysed and validated the data, critically reviewed and revised the manuscript. All authors approved the final version as submitted and agree to be accountable for all aspects of the work, ensuring its accuracy and integrity.

## Conflicts of Interest

Pfeiffer N received financial support and grants from Novartis, Ivantis, Santen, Thea, Boehringer Ingelheim Deutschland GmbH & Co. KG, Alcon and Sanoculis. Schuster AK received research support from Allergan, Bayer, Heidelberg Engineering, PlusOptix and Novartis. Fieß A, Hartmann A, Mildenberger E, Wackernagel D, Winter J, Lackner KJ, Gißler S: none.

## Supporting information


**Appendix S1:** Supporting information.

## Data Availability

Data are available upon reasonable request. Interested researchers should make their requests to the coordinating PI of the Gutenberg Prematurity Study (Achim Fieß; achim.fiess@unimedizin-mainz.de).
