# Supplementary material for: Preterm Birth, Fetal Growth Restriction and Early Postnatal Body Mass Index Normalisation Predict Adult Anthropometry
Source: Acta Paediatr. 2025 Nov 12;115(3):623–33. doi: 10.1111/apa.70368 (PMC12890003; doi:10.1111/apa.70368)
Supplement: Supplementary file 1 — Appendix S1: Supporting information. [file APA-115-623-s001.pdf]

Table S1. Analysis of a potential effect modification between birth weight percentile groups and gestational age.

	All models adjusted for age and sex.	
**Body height [cm]**		
**Linear regression**	**Estimate (95% CI)**	**p-value**
Gestational age deficit (weeks)	-5.475<br>(-0.559, -0.264)	<0.001
Severely SGA (<3)	-1.795<br>(-5.930, 0.267)	0.07
Moderately SGA (3 to <10)	-3.034<br>(-6.491, -1.390)	0.003
Moderately LGA (>90 to 97)	1.579<br>(-0.466, 4.295)	0.11
Severely LGA (>97)	2.755<br>(0.952, 5.685)	0.01
Gestational age deficit* Severely SGA (<3)	-2.140<br>(-1.280, -0.055)	0.03
Gestational age deficit* Moderately SGA (3 to <10)	0.496<br>(-0.302, 0.506)	0.62
Gestational age deficit * Moderately LGA (>90 to 97)	-0.042<br>(-0.891, 0.854)	0.97
Gestational age deficit * Severely LGA (>97)	0.357<br>(-1.119, 1.615)	0.72
**Body weight [kg]**		
**Quantile regression**	**$\beta_{(\tau_{50})}$ [95%-CI]**	**p-value**
Gestational age deficit (weeks)	-0.391<br>(-0.889, -0.143)	0.05
Severely SGA (<3)	-3.702<br>(-8.646, -0.735)	0.15
Moderately SGA (3 to <10)	-4.487<br>(-10.229, 0.028)	0.13
Moderately LGA (>90 to 97)	3.044<br>(-2.811, 8.918)	0.48
Severely LGA (>97)	7.318<br>(0.745, 11.413)	0.01
Gestational age deficit* Severely SGA (<3)	-0.553<br>(-1.189, 0.794)	0.29

Gestational age deficit* Moderately SGA (3 to <10)	-0.132 (-0.712, 0.653)	0.78
Gestational age deficit * Moderately LGA (>90 to 97)	3.956 (0.139, 7.168)	0.05
Gestational age deficit * Severely LGA (>97)	-1.770 (-2.519, 2.925)	0.26
**Body-mass-index**		
**Quantile regression**	**$\beta_{(\tau 50)}$ [95%-CI]**	**p-value**
Gestational age deficit (weeks)	-0.035 (-0.089, 0.102)	0.48
Severely SGA (<3)	0.098 (-0.089, 0.102)	0.90
Moderately SGA (3 to <10)	0.059 (-1.12, 1.870)	0.95
Moderately LGA (>90 to 97)	0.245 (-0.418, 2.110)	0.76
Severely LGA (>97)	1.440 (-0.235, 2.256)	0.06
Gestational age deficit* Severely SGA (<3)	-0.080 (-0.588, 0.509)	0.74
Gestational age deficit* Moderately SGA (3 to <10)	-0.116 (-0.278, 0.091)	0.36
Gestational age deficit * Moderately LGA (>90 to 97)	0.523 (0.067, 2.359)	0.57
Gestational age deficit * Severely LGA (>97)	-0.216 (-1.381, 0.912)	0.68
**Head circumference**		
**Linear regression**	**Estimate (95% CI)**	**p-value**
Gestational age deficit (weeks)	-0.059 (-0.127, 0.008)	0.08
Severely SGA (<3)	-0.508 (-1.944, 0.928)	0.49
Moderately SGA (3 to <10)	0.251 (-0.914, 1.417)	0.67
Moderately LGA (>90 to 97)	1.050 (-0.038, 2.138)	0.06
Severely LGA (>97)	1.325 (0.233, 2.418)	0.02
Gestational age deficit* Severely SGA (<3)	-0.106 (-0.388, 0.176)	0.46

Gestational age deficit* Moderately SGA (3 to <10)	-0.113<br>(-0.298, 0.071)	0.23
Gestational age deficit * Moderately LGA (>90 to 97)	0.029<br>(-0.370, 0.427)	0.89
Gestational age deficit * Severely LGA (>97)	0.094<br>(-0.531, 0.719)	0.77

Legend: Gestational age deficit represents the number of weeks by which the gestation is shorter than the standard term pregnancy of 40 weeks. SGA - small for gestational age; LGA - large for gestational age. Reference for SGA/LGA comparisons: AGA - appropriate for gestational age.

Table S2. Association analyses of the anthropometric parameters for adults born preterm and term (n = 610)

	All models adjusted for age, sex	
**Body height [cm]**		
**Linear regression**	**Estimate (95% CI)**	**p-value**
Gestational age deficit (weeks)	-0.365<br>(-0.488,-0.242)	<0.001
Birth weight percentile	0.074<br>(0.057, 0.091)	<0.001
**Body weight [kg]**		
**Quantile regression**	**$\beta_{(\tau 50)}$ (95%-CI)**	**p-value**
Gestational age deficit (weeks)	-0.386<br>(-0.624, -0.183)	0.01
Birth weight percentile	0.113<br>(0.113, 0.082)	<0.001
**Body-mass-index (BMI)**		
**Quantile regression**	**$\beta_{(\tau 50)}$ (95%-CI)**	**p-value**
Gestational age deficit (weeks)	-0.031<br>(-0.084, 0.045)	0.43
Birth weight percentile	0.010<br>(0.004, 0.018)	0.05
**Head circumference**		
**Linear regression**	**Estimate (95% CI)**	**p-value**
Gestational age deficit (weeks)	-0.112<br>(-0.147, -0.076)	<0.001
Birth weight percentile	0.016<br>(0.011, 0.021)	<0.001

* Gestational age deficit represents the number of weeks by which the gestation is shorter than the standard term pregnancy of 40 weeks.

Table S3. Association analysis for metabolic parameters in adulthood for individuals born preterm and at term

	All models adjusted for age and sex.	
**Linear regression**	**Estimate (95% CI)**	**p-value**
**Cholesterol [mg/dL]**		
Gestational age deficit (weeks)	-0.151 (-0.871, 0.569)	0.68
Severely SGA (<3)	1.770 (-9.023, 12.562)	0.75
Moderately SGA (3 to <10)	-3.497 (-12.699, 5.704)	0.46
Moderately LGA (>90 to 97)	-3.337 (-16.347, 9.672)	0.61
Severely LGA (>97)	-5.356 (-19.342, 8.630)	0.45
**Fasting glucose [mg/dL]**		
Gestational age deficit (weeks)	-0.300 (-0.871, 0.272)	0.30
Severely SGA (<3)	-2.096 (-10.638, 6.447)	0.63
Moderately SGA (3 to <10)	1.202 (-6.124, 8.527)	0.75
Moderately LGA (>90 to 97)	-5.846 (-16.230, 4.518)	0.27
Severely LGA (>97)	-7.158 (-17.937, 3.621)	0.19
**Triglycerides [mg/dL])**		
Gestational age deficit (weeks)	0.163 (-1.067, 1.392)	0.80
Severely SGA (<3)	7.471 (-10.956, 25.897)	0.43
Moderately SGA (3 to <10)	8.667 (-7.127, 24.459)	0.28
Moderately LGA (>90 to 97)	-7.490 (-29.703, 14.722)	0.51

Severely LGA (>97)	-4.244<br>(-28.123, 19.636)	0.73
**Creatinine [mg/dL]**		
Gestational age deficit (weeks)	-0.001<br>(-0.004, 0.003)	0.74
Severely SGA (<3)	0.02<br>(-0.028, 0.063)	0.44
Moderately SGA (3 to <10)	0.003<br>(-0.036, 0.041)	0.90
Moderately LGA (>90 to 97)	0.031<br>(-0.024, 0.086)	0.27
Severely LGA (>97)	-0.070<br>(-0.127, -0.013)	0.02

Legend: Gestational age deficit represents the number of weeks by which the gestation is shorter than the standard term pregnancy of 40 weeks. SGA - small for gestational age; LGA - large for gestational age. Reference for SGA/LGA comparisons: AGA - appropriate for gestational age.

Table S4. Association analysis for risk of hypertension and metabolic syndrome in adulthood for individuals born preterm and at term.

	All models adjusted for age and sex.	
**Multivariable logistic regression**	**OR**	**p-value**
**Hypertension***		
Gestational age deficit (weeks)	1.013 (0.968, 1.060)	0.55
Severely SGA (<3)	0.925 (0.4870, 1.699)	0.81
Moderately SGA (3 to <10)	0.631 (0.341, 1.124)	0.13
Moderately LGA (>90 to 97)	0.816 (0.365, 1.747)	0.61
Severely LGA (>97)	0.768 (0.335, 1.675)	0.52
**Metabolic syndrome**		
Gestational age deficit (weeks)	1.002 (0.938, 1.069)	0.95
Severely SGA (<3)	0.718 (0.259, 1.699)	0.48
Moderately SGA (3 to <10)	0.429 (0.142, 1.054)	0.09
Moderately LGA (>90 to 97)	0.912 (0.305, 2.394)	0.86
Severely LGA (>97)	0.869 (0.290, 2.296)	0.79

Legend: Gestational age deficit represents the number of weeks by which the gestation is shorter than the standard term pregnancy of 40 weeks. SGA - small for gestational age; LGA - large for gestational age. Reference for SGA/LGA comparisons: AGA - appropriate for gestational age. Arterial hypertension was defined by antihypertensive medication use, systolic blood pressure >140 mmHg, diastolic blood pressure >90 mmHg, or an established diagnosis. Metabolic syndrome was defined by NCEP-ATP III criteria("Third Report of the National Cholesterol Education Program (NCEP) Expert Panel on Detection, Evaluation, and Treatment of High Blood Cholesterol in Adults (Adult Treatment Panel III) final report," 2002): abdominal obesity (waist >102 cm in men, >88 cm in women), triglycerides ≥150 mg/dL, HDL <40 mg/dL in men (<50 mg/dL in women), blood pressure ≥135/85 mmHg, and fasting glucose >110 mg/dL or type 2 diabetes.

Table S5. Association analyses of the anthropometric parameters for adults born preterm and term adjusted for maternal anthropometry.

	All models adjusted for age, sex	
**Body height [cm]**		
**Linear regression**	**Estimate (95% CI)**	**p-value**
Maternal height	0.405<br>(0.275, 0.535)	<0.001
Gestational age deficit*<br>(weeks)	-0.392<br>(-0.577, -0.207)	<0.001
Severely SGA<br>(<3)	-5.360<br>(-7.800, -2.921)	<0.001
Moderately SGA<br>(3 to <10)	-2.860<br>(-5.344, -0.376)	0.02
Moderately LGA<br>(>90 to 97)	-0.214<br>(-3.334, 2.906)	0.89
Severely LGA<br>(>97)	-0.149<br>(-3.342, 3.044)	0.93
**Body weight [kg]**		
**Quantile regression**	**$\beta_{(\tau 50)}$ [95%-CI]**	**p-value**
Maternal weight	0.155<br>(0.108, 0.255)	0.004
Gestational age deficit (weeks)	-0.122<br>(-0.521, 0.307)	0.60
Severely SGA<br>(<3)	-8.457<br>(-10.823, -4.556)	<0.001
Moderately SGA<br>(3 to <10)	-7.702<br>(-11.606, -3.398)	0.01
Moderately LGA<br>(>90 to 97)	5.408<br>(-0.871, 9.545)	0.13
Severely LGA<br>(>97)	1.359<br>(-1.480, 7.425)	0.64
**Body-mass-index**		
**Quantile regression**	**$\beta_{(\tau 50)}$ [95%-CI]**	**p-value**
Maternal BMI	0.191<br>(0.090, 0.295)	0.001
Gestational age deficit<br>(weeks)	0.005<br>(-0.060, 0.112)	0.92
Severely SGA<br>(<3)	-1.813<br>(-2.690, 0.205)	0.01
Moderately SGA<br>(3 to <10)	-2.154<br>(-3.044, -0.808)	<0.001
Moderately LGA<br>(>90 to 97)	0.473<br>(-1.525, 3.509)	0.61
Severely LGA	-0.727	0.48

(>97)	(-1.631, 1.242)	

* Gestational age deficit represents the number of weeks by which the gestation is shorter than the standard term pregnancy of 40 weeks. SGA - small for gestational age; LGA - large for gestational age. Reference for SGA/LGA comparisons: AGA - appropriate for gestational age.

Table S6. Association analyses of the anthropometric parameters with percentile change between birth and six months of age for adults born small for gestational age and at preterm.

	All multivariable models adjusted for age, sex	
**Body height [cm]**		
**Linear regression**	**Estimate (95% CI)**	**p-value**
Percentile change up to six months	-0.387 (-9.972, 9.198)	0.93
Gestational age deficit (weeks)	-0.810 (-1.678, 0.057)	0.07
Severely SGA (<3)	3.913 (-8.913, 1.087)	0.12
**Body weight [kg]**		
**Quantile regression**	**$\beta_{(\tau_{50})}$ [95%-CI]**	**p-value**
Percentile change up to six months	14.493 (10.708, 45.304)	0.43
Gestational age deficit (weeks)	-0.189 (-0.626, 1.338)	0.85
Severely SGA (<3)	0.797 (-3.656, 11.072)	0.89
**Body-mass-index**		
**Quantile regression**	**$\beta_{(\tau_{50})}$ [95%-CI]**	**p-value**
Percentile change up to six months	3.780 (0.085, 10.496)	0.52
Gestational age deficit (weeks)	0.084 (-0.228, 0.529)	0.73
Severely SGA (<3)	0.681 (-1.007, 3.698)	0.71
**Head circumference**		
**Linear regression**	**Estimate (95% CI)**	**p-value**
Percentile change up to six months	3.787 (1.652, 5.922)	0.001
Gestational age deficit (weeks)	-0.148 (-0.341, 0.045)	0.13
Severely SGA (<3)	-0.861 (-1.974, 0.253)	0.12

* Gestational age deficit represents the number of weeks by which the gestation is shorter than the standard term pregnancy of 40 weeks. SGA - small for gestational age. Reference for SGA comparisons: SGA (3 to >10 percentile)

Table S7. Association analyses of the anthropometric parameters with percentile change between birth and six months of age for adults born small for gestational age and at term.

	Multivariable model adjusted for age, sex	
**Body height [cm]**		
**Linear regression**	**Estimate (95% CI)**	**p-value**
Percentile change up to six months	-8.262<br>(-15.865, -0.658)	0.03
Gestational age deficit (weeks)	0.004<br>(-1.288, 1.296)	0.99
Severely SGA (<3)	1.196<br>(-2.212, 4.606)	0.48
**Body weight [kg]**		
**Quantile regression**	**$\beta_{(\tau 50)}$ [95%-CI]**	**p-value**
Percentile change up to six months	-0.174<br>(-11.573, 7.859)	0.97
Gestational age deficit (weeks)	-0.587<br>(-2.728, 1.253)	0.65
Severely SGA (<3)	0.129<br>(-6.282, 5.167)	0. .97
**Body-mass-index**		
**Quantile regression**	**$\beta_{(\tau 50)}$ [95%-CI]**	**p-value**
Percentile change up to six months	0.975<br>(-3.347, 6.487)	0.72
Gestational age deficit (weeks)	-0.366<br>(-0.984, 0.572)	0.49
Severely SGA (<3)	0.278<br>(-4.925, 2.466)	0.85
**Head circumference**		
**Linear regression**	**Estimate (95% CI)**	**p-value**
Percentile change up to six months	0.281<br>(-1.851, 2.412)	0.79
Gestational age deficit (weeks)	0.040<br>(-0.323, 0.402)	0.83
Severely SGA (<3)	-0.532<br>(-1.505, 0.441)	0.28

* Gestational age deficit represents the number of weeks by which the gestation is shorter than the standard term pregnancy of 40 weeks. SGA - small for gestational age. Reference for SGA comparisons: SGA (3 to >10 percentile)

Supplemental Figure 1. 3D surface plots of a) body height [cm], b) body weight [kg], c) body-mass-index and d) head circumference [cm] stratified by birth weight percentile groups in participants born preterm and at term.

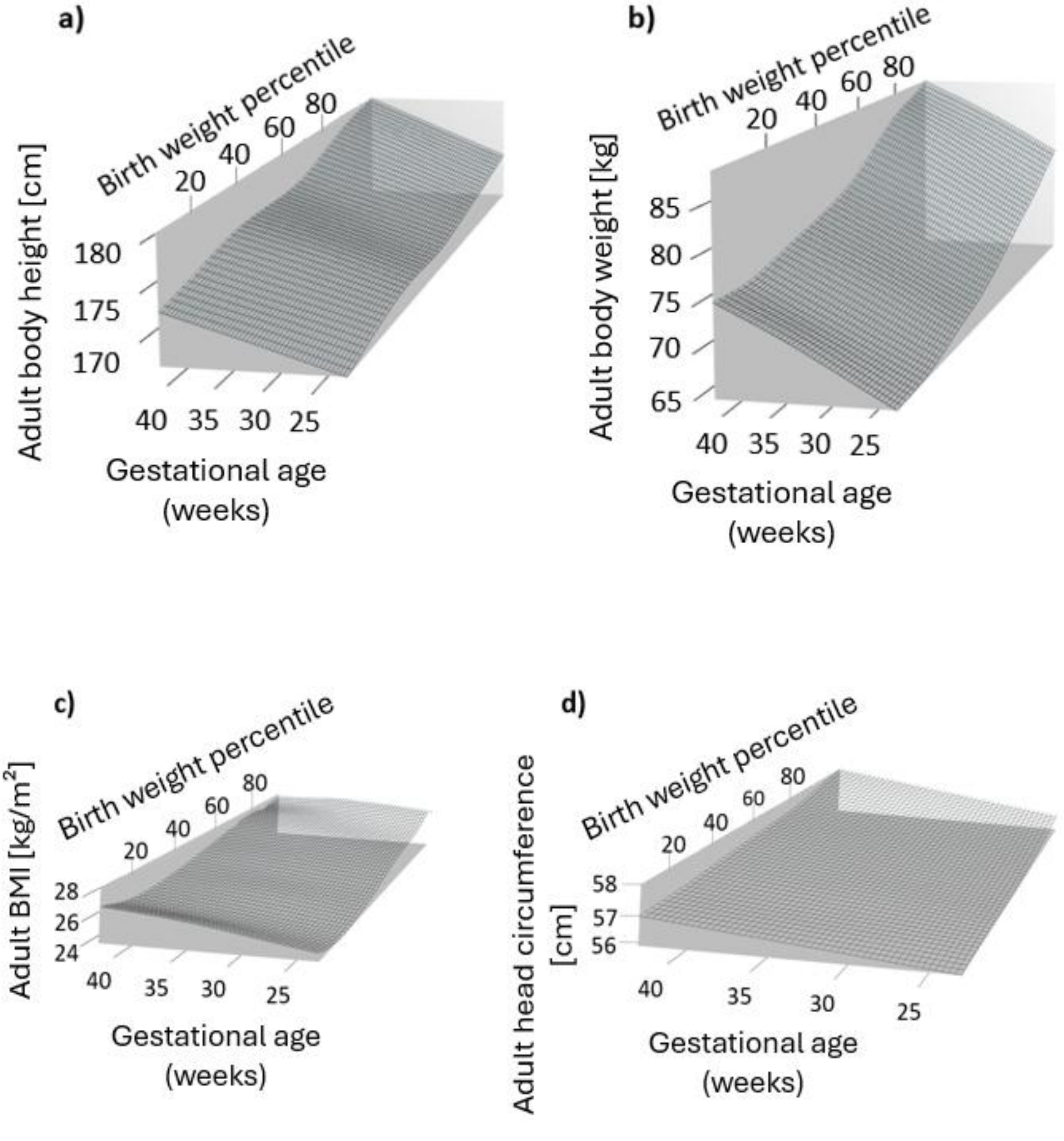

Supplemental Figure 2. 3D bar charts of a) body height [cm], b) body weight [kg], c) head circumference [cm], and d) BMI [kg/m²] stratified by birth weight percentile groups and gestational age (weeks) (n = 606).

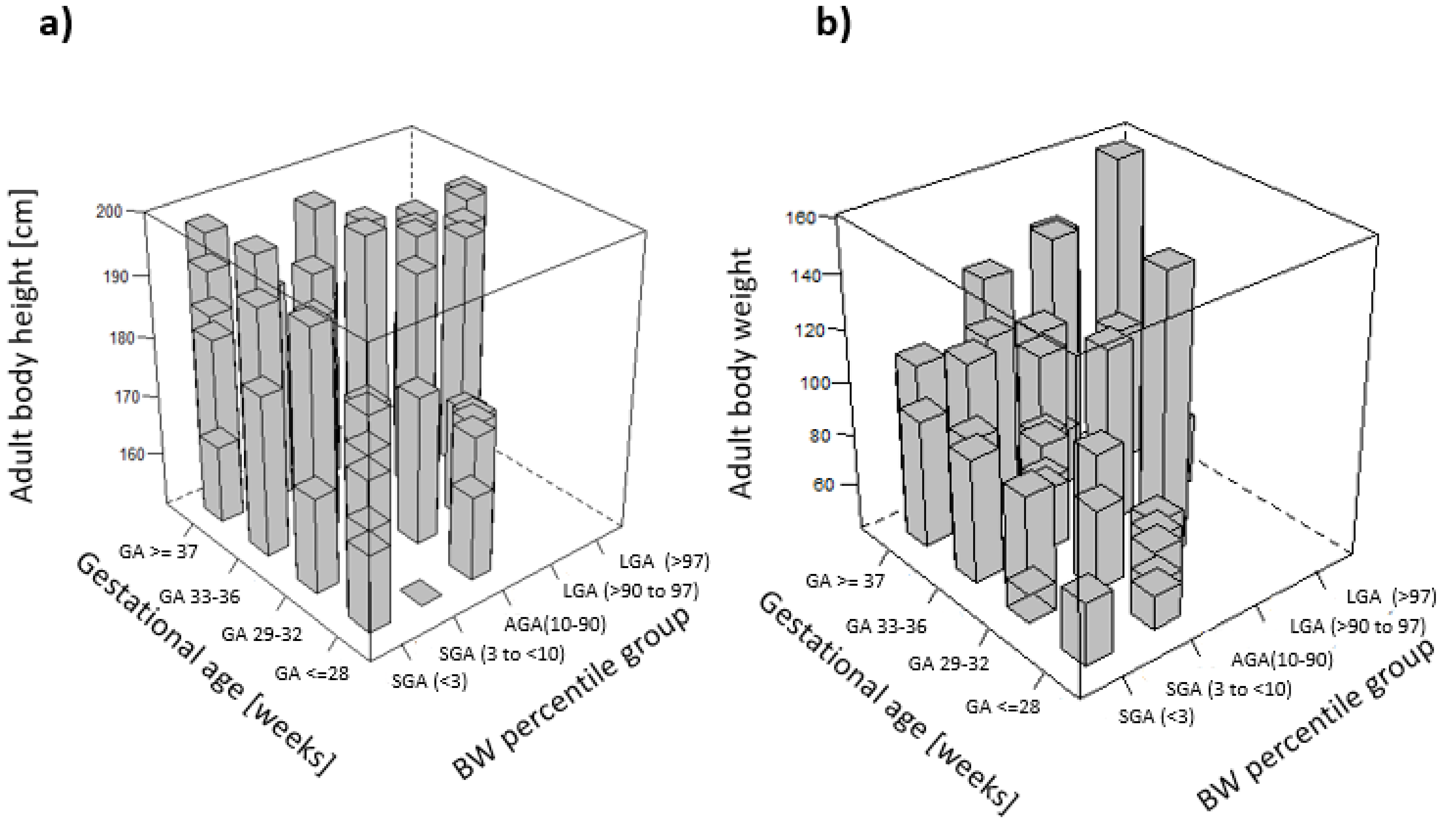

c) d)

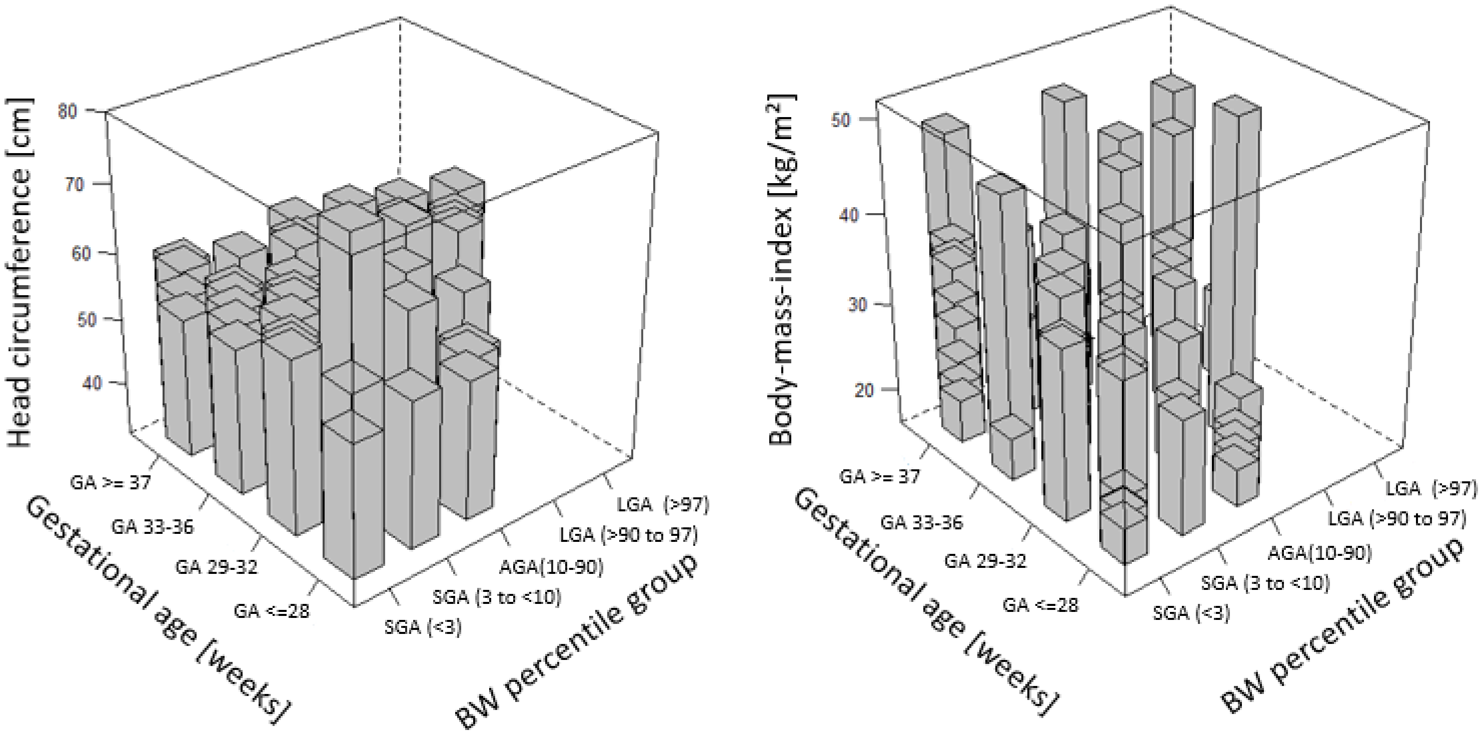
Head circumference [cm]
80
70
60
50
40
GA >= 37
GA 33-36
GA 29-32
GA <=28
Gestational age [weeks]
LGA (>97)
LGA (>90 to 97)
AGA(10-90)
SGA (3 to <10)
SGA (<3)
BW percentile group
Body-mass-index [kg/m²]
50
40
30
20
GA >= 37
GA 33-36
GA 29-32
GA <=28
Gestational age [weeks]
LGA (>97)
LGA (>90 to 97)
AGA(10-90)
SGA (3 to <10)
SGA (<3)
BW percentile group

Supplemental Figure 3. A: Boxplots for participants born preterm and severe or moderately small for gestational age (SGA) of a) body height [cm], b) body weight [kg], c) BMI [kg/m²], and d) head circumference [cm] stratified by achievement of normalisation of postnatal body mass index percentiles. B: Boxplots for participants born term (gestational age ≥37 weeks) and severely or moderately SGA of a) body height [cm], b) body weight [kg], c) BMI [kg/m²], d) head circumference [cm] stratified by the achievement of catch-up-growth.

A

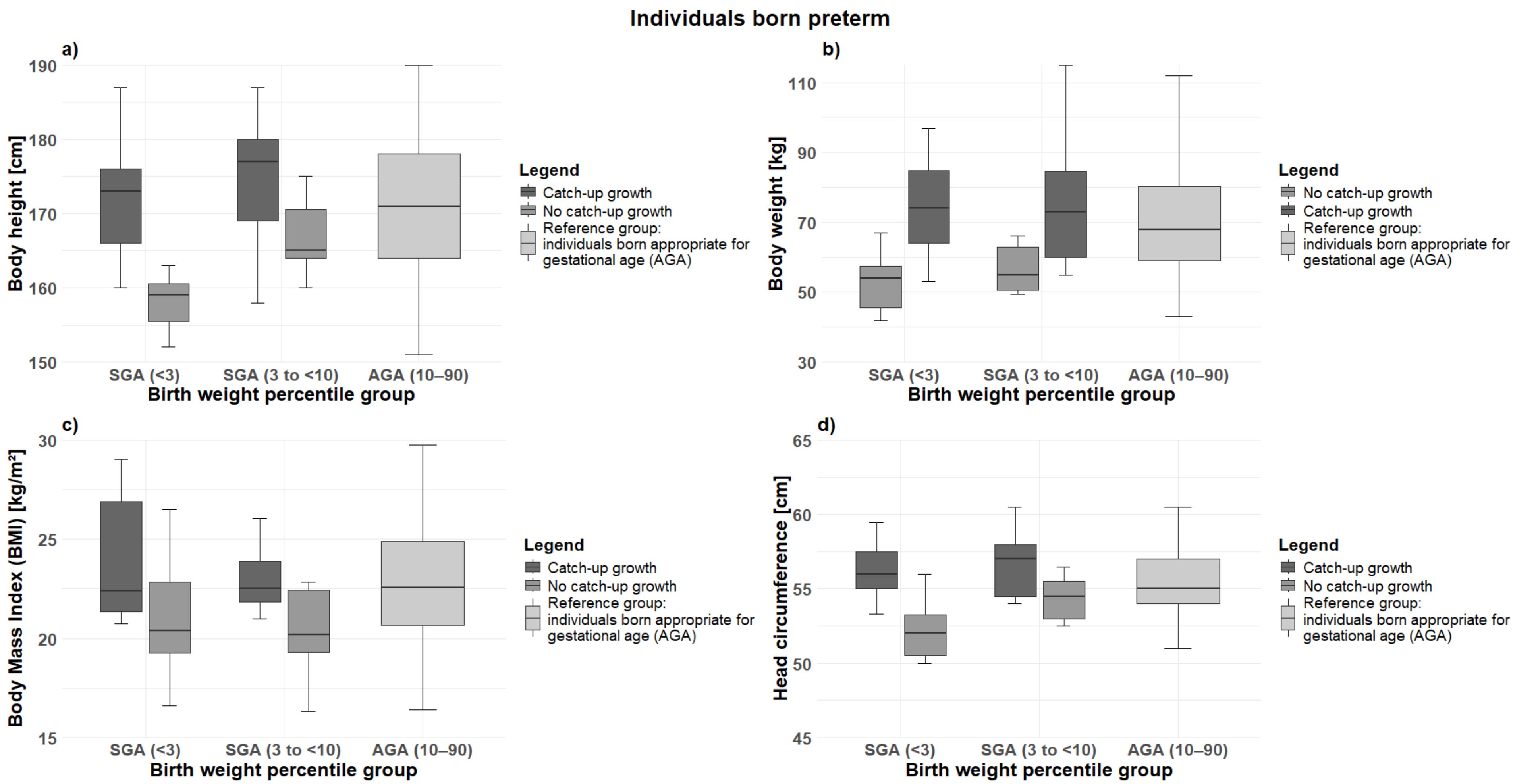

Individuals born preterm
a)
Body height [cm]
190
180
170
160
150
SGA (<3)
SGA (3 to <10)
AGA (10–90)
Birth weight percentile group
Legend
Catch-up growth
No catch-up growth
Reference group: individuals born appropriate for gestational age (AGA)
b)
Body weight [kg]
110
90
70
50
30
SGA (<3)
SGA (3 to <10)
AGA (10–90)
Birth weight percentile group
Legend
No catch-up growth
Catch-up growth
Reference group: individuals born appropriate for gestational age (AGA)
c)
Body Mass Index (BMI) [kg/m²]
30
25
20
15
SGA (<3)
SGA (3 to <10)
AGA (10–90)
Birth weight percentile group
Legend
Catch-up growth
No catch-up growth
Reference group: individuals born appropriate for gestational age (AGA)
d)
Head circumference [cm]
65
60
55
50
45
SGA (<3)
SGA (3 to <10)
AGA (10–90)
Birth weight percentile group
Legend
Catch-up growth
No catch-up growth
Reference group: individuals born appropriate for gestational age (AGA)

B

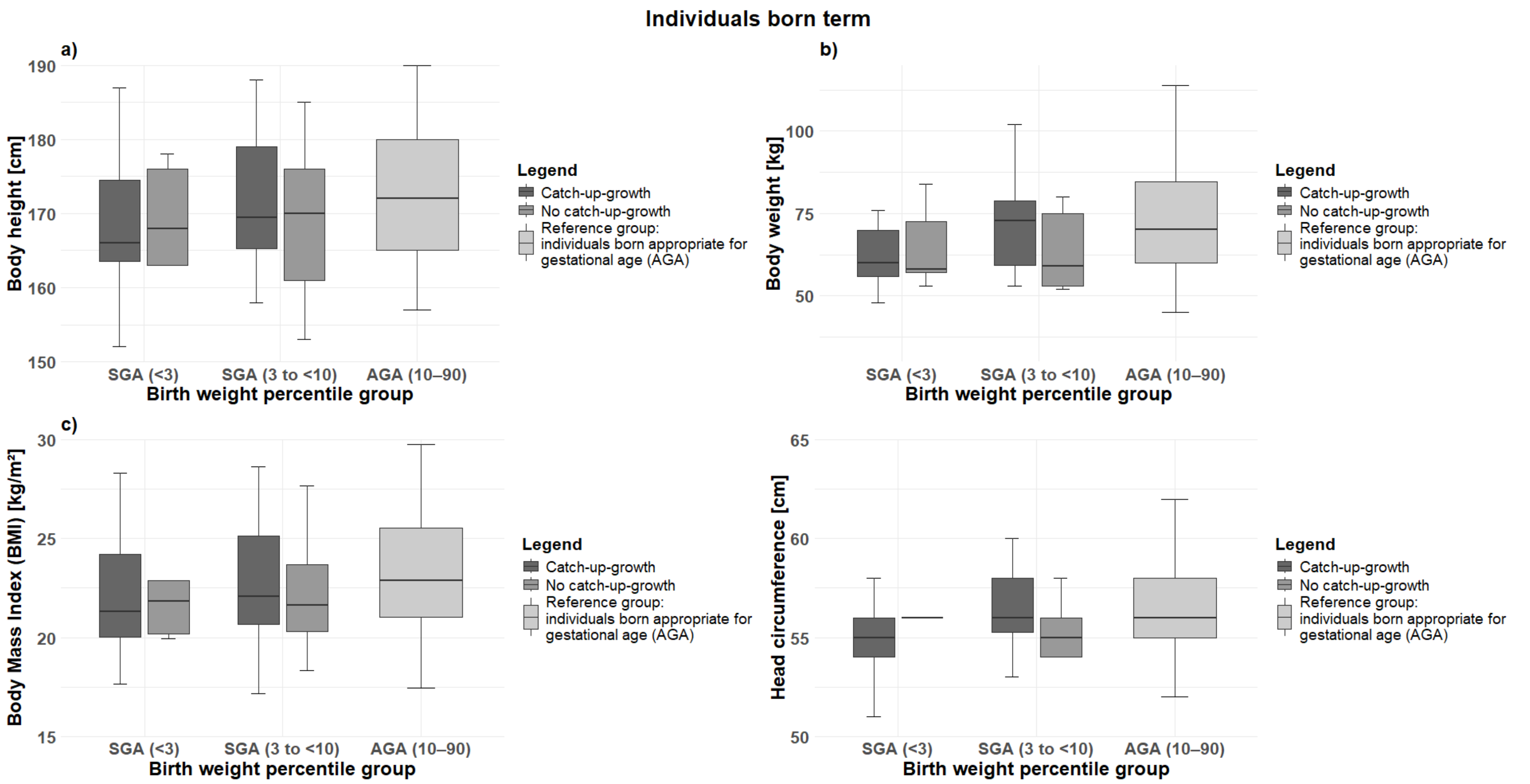
Individuals born term
a)
Body height [cm]
190
180
170
160
150
SGA (<3)
SGA (3 to <10)
AGA (10–90)
Birth weight percentile group
Legend
Catch-up-growth
No catch-up-growth
Reference group:
individuals born appropriate for
gestational age (AGA)
b)
Body weight [kg]
100
75
50
SGA (<3)
SGA (3 to <10)
AGA (10–90)
Birth weight percentile group
Legend
Catch-up-growth
No catch-up-growth
Reference group:
individuals born appropriate for
gestational age (AGA)
c)
Body Mass Index (BMI) [kg/m²]
30
25
20
15
SGA (<3)
SGA (3 to <10)
AGA (10–90)
Birth weight percentile group
Legend
Catch-up-growth
No catch-up-growth
Reference group:
individuals born appropriate for
gestational age (AGA)
Head circumference [cm]
65
60
55
50
SGA (<3)
SGA (3 to <10)
AGA (10–90)
Birth weight percentile group
Legend
Catch-up-growth
No catch-up-growth
Reference group:
individuals born appropriate for
gestational age (AGA)
